# Post-treatment Stability of Maxillary Arch Dimensions in Unilateral Cleft Lip and Palate: A Systematic Review

**DOI:** 10.7759/cureus.101175

**Published:** 2026-01-09

**Authors:** Ahmed A Alasmari, Ghadi M Alshahrani, Amjad Mohammad Alasmari, Abdulrahman Saad A. Alasmari, Amwaj Awn Alqahtani, Abdullah Tariq Almalki, Mohammed Dhafer Alqahtani, Sarah Jubran Alqahtani, Atheer Hadi A. Almukawwi, Nada Saad Alshahrani, Omar Saeed Alshahrani

**Affiliations:** 1 Orthodontics and Dentofacial Orthopaedics, Armed Forces Hospital Southern Region, Khamis Mushait, SAU; 2 Dentistry, King Khalid University, Abha, SAU; 3 General Dentistry, King Khalid University, Abha, SAU; 4 General Dentistry, Almalki Dental Clinic, Abha, SAU; 5 Pediatric Dentistry, Armed Forces Hospital Southern Region, Khamis Mushait, SAU

**Keywords:** cleft lip and palate, maxillary arch, orthodontic retention, relapse, unilateral cleft lip and palate

## Abstract

Cleft lip and palate (CLP) are structural anomalies resulting from the failure of craniofacial prominences to fuse during embryonic development. Orthodontic treatment seeks to address these conditions by improving dentofacial balance and restoring oral function, but long-term post-treatment stability continues to pose a challenge. This systematic review included a literature search of four electronic databases (PubMed, Scopus, Web of Science, and Cochrane CENTRAL) to identify observational evidence on the stability of orthodontic treatment in CLP. Six studies, comprising three cohort studies and three case reports, were included, totaling 130 participants (110 individuals with CLP, including 54 males and 56 females, and 20 non-cleft controls).

Cohort evidence suggested that maxillary arch form may influence post-treatment stability, with symmetrical arches demonstrating minimal relapse, whereas arches with anterior collapse or combined segment collapse showed a higher tendency toward relapse. Across cohort studies, maxillary arch dimensions generally decreased during follow-up regardless of retainer or prosthesis use. Fixed partial dentures appeared to offer better stability for anterior measurements than implant-supported prostheses, while greater instability was consistently observed in the molar region. Case reports described appropriate orthodontic approaches in complex cleft presentations, with variable reporting of adverse events, and also reported stable transplanted teeth without evidence of ankylosis or root resorption. Overall, stability outcomes may be influenced by the retention strategy, arch form severity, and prosthesis type and duration. Further well-designed observational studies with standardized stability outcomes and consistent follow-up intervals are needed to identify the best approaches to enhance long-term stability.

## Introduction and background

Orofacial clefts are one of the most prevalent craniofacial congenital anomalies and can affect the lip with or without involvement of the palate, or involve the palate alone. According to the World Health Organization, the global prevalence estimate for cleft lip and palate (CLP) is approximately one child in every 700 births [1]. The exact etiology of CLP is unknown, but it is multifactorial and primarily influenced by environmental and genetic factors [2,3]. Compared to the general population, patients with CLP are more commonly affected by dental anomalies, such as tooth agenesis, tooth impaction on the cleft side, and morphological irregularities. These anomalies can affect facial appearance, functional occlusion, and speech development [4,5].

The treatment and rehabilitation process for patients with CLP is long and complex, spanning from infancy to early adulthood. Care is provided from birth to adulthood, and intervention occurs across several phases corresponding to the stages of facial and dental development [6]. The treatment protocol encompasses several key milestones: surgical lip repair is typically performed in the first six months after birth, with soft palate closure recommended during the first year of life, and hard palate repair planned based on maxillary growth considerations. Secondary alveolar bone grafting is performed to close the alveolar cleft, with timing based on the position and root formation stage of the maxillary canine; this can be supplemented with tertiary bone grafting in adulthood if needed [7]. Accordingly, orthodontic treatment is essential for patients with CLP to address dentoalveolar disorders, malinclined teeth, crossbite, and crowding, and thereby improve patients’ quality of life [8]. Patient needs dictate the specific combination of appliances and techniques used in treatment.

Several studies have investigated post-treatment outcomes associated with orthodontic treatment in patients with CLP. Orthodontic treatment has been shown to improve dental alignment, facial aesthetics, occlusion [9-11], and oral function [12,13]. However, ensuring the long-term stability of these treatment outcomes remains a significant challenge, and relapse is common among patients with CLP after orthodontic treatment [14]. Previous studies have reported variable findings regarding maxillary arch stability. For instance, Venkateshwaran et al. reported that slow maxillary expansion (SME) is less traumatic but may be associated with greater disruption of post-expansion stability than rapid maxillary expansion (RME) [15]. Rasool et al. indicated that SME may require a shorter retention period and offer better maintenance than rapid palatal expansion [16]. Caballero et al. compared the stability of orthodontic treatment in patients with CLP and non-cleft patients, and reported variable findings in maxillary arch distances. Intercanine distance (IC) was stable in the cleft group rehabilitated with a fixed partial denture (FPD) but unstable in the control group. The interfirst premolar distance was unstable in the cleft group with FPD but stable in the non-cleft group, whereas both the cleft group with FPD and the non-cleft group showed a reduction in intermolar distance (IM) [17].

Post-treatment stability in patients with CLP can be achieved using orthodontic retainers, circumferential fiberotomy, and overcorrection. The factors influencing relapse include treatment type, cleft severity, and previous complications. Studies often focus on relapse and long-term dental changes without adequately considering the type of retention and rehabilitation required for stability. The primary objective of this systematic review was to investigate the stability of maxillary arch dimensions and relapse patterns after orthodontic treatment in patients with CLP. It also aimed to examine the effects of different retention protocols, prosthetic rehabilitation strategies, and patient-related factors on long-term treatment stability.

## Review

Methods

Protocol Design

This systematic review was conducted in accordance with the Cochrane Handbook for Systematic Reviews [18] and reported based on the PRISMA (Preferred Reporting Items for Systematic Reviews and Meta-Analyses) 2020 checklist [19]. Although orthodontic treatment plays an essential role in rehabilitating patients with CLP, post-treatment stability remains a major clinical concern. Relapse may occur due to intrinsic maxillary growth deficiency, scar tissue from previous surgeries, and the absence of bony support in the cleft area. Clinically, instability is often reflected by narrowing of the maxillary arch, reopening of spaces in the cleft region, recurrence of crossbites, rotation or displacement of anterior teeth, and progressive changes in intercanine or intermolar width. These changes may compromise esthetics, masticatory efficiency, prosthetic rehabilitation, and patient satisfaction.

Despite the importance of maintaining long-term stability, the existing literature is fragmented, often focuses on small samples, and uses variable follow-up intervals and outcome measures. Many studies describe treatment techniques but provide limited information about relapse patterns, retention strategies, or the long-term effectiveness of these approaches. Therefore, a systematic synthesis of the available observational evidence is needed to clarify stability outcomes and identify clinical factors associated with relapse in cleft populations.

The protocol was developed prospectively to examine orthodontic treatment stability in patients with complete unilateral CLP (UCLP) using a comprehensive PICO (Patient/Problem, Intervention, Comparison, and Outcome) framework. The population comprised non-syndromic individuals with UCLP, ranging from four years old to young adulthood (≤30 years), who had completed primary cleft surgery (lip closure at 3-12 months and palate closure at 10-24 months) before orthodontic intervention. Contemporary orthodontic approaches used for cleft rehabilitation were evaluated, including SME protocols utilizing light forces over extended periods (up to two years), RME using mechanical or hydraulic expanders, and comprehensive fixed multibracket appliance therapy with edgewise or standard bracket systems. Ancillary techniques, including tooth extraction, autotransplantation, and mini-implant anchorage, were also eligible. Treatment durations ranged from variable presurgical periods to approximately two to more than eight years for comprehensive orthodontic management.

Comparators primarily consisted of within-study variations rather than formal parallel comparison groups, including different retention protocols (bonded retainers, circumferential retainers, fixed prosthetic retention, or no retention), inherent arch morphology variations (symmetrical versus collapsed arch forms), and post-orthodontic prosthetic rehabilitation modalities (dental implants versus fixed partial dentures). Where available, non-cleft control groups provided reference data for comparative stability assessment. The primary outcomes focused on the quantitative stability of maxillary dental arch dimensions measured using digitized dental casts or three-dimensional stereophotogrammetry, assessed at a minimum of nine months (0.75 years) post-treatment, with follow-up extending beyond eight years.

Key measurements included IC, IM, interpremolar distance (IPM), and sagittal arch length (SA). Eligible study designs included prospective and retrospective cohort studies with a mean follow-up duration of ≥1 year, case-control studies, and case reports with ≥1 year of documented follow-up. Studies were limited to English-language, peer-reviewed publications with no restrictions on publication date. Exclusion criteria included studies involving syndromic clefts (genetic syndromes with a cleft component), isolated cleft palate without lip involvement, incomplete clefts, or cases combined with orthognathic surgery. In addition, secondary literature (systematic reviews and meta-analyses), studies lacking adequate outcome data, cross-sectional studies, conference abstracts or proceedings, and study protocols without reported results were excluded.

Eligibility Criteria

Studies were eligible if they included non-syndromic individuals with complete UCLP who had completed primary cleft surgery before orthodontic intervention. Eligible interventions were contemporary orthodontic approaches used in cleft rehabilitation, with or without adjunctive procedures such as extraction, autotransplantation, or skeletal anchorage. Studies were required to report post-treatment stability outcomes for maxillary arch dimensions, including IC, IM, IPM, and SA, assessed at a minimum of nine months (0.75 years) after treatment completion. Eligible study designs included prospective and retrospective cohort studies with a mean follow-up duration of ≥1 year, case-control studies, and case reports with ≥1 year of documented follow-up. Studies were restricted to peer-reviewed English-language publications with no date limitations.

Exclusion criteria were established to reduce confounding factors and enhance comparability among studies. Studies involving syndromic clefts were excluded because syndrome-related systemic anomalies and growth disturbances may independently affect orthodontic outcomes and relapse patterns. Isolated cleft palate without lip involvement and incomplete clefts were also excluded, as these conditions differ from complete unilateral cleft lip and palate with respect to surgical approaches, growth characteristics, and orthodontic management. Studies including patients who underwent concurrent orthognathic surgery were excluded because skeletal repositioning modifies maxillary anatomy and would confound evaluation of orthodontic stability alone. Studies lacking adequate outcome data, such as missing arch measurements or insufficient follow-up, were excluded because reliable assessment of stability was not possible. Finally, secondary literature (systematic reviews and meta-analyses), cross-sectional studies, conference abstracts, and study protocols without reported results were excluded due to the absence of original longitudinal clinical data required to assess post-treatment stability.

Information Sources and Search Timing

The electronic search was conducted across four databases: PubMed, Scopus, Web of Science, and Cochrane CENTRAL. The search strategy was adapted to the requirements and syntax of each database. No restrictions on publication date were imposed, and the final search was conducted on January 17, 2026. Reference lists of the included studies were also reviewed to identify additional eligible articles not retrieved through the database search.

Study Selection Process

All retrieved records were exported into a reference management system for deduplication. Two independent reviewers screened titles and abstracts to identify potentially eligible studies. Full-text articles were then retrieved and assessed independently by the same reviewers against the predefined inclusion and exclusion criteria. Disagreements at any stage were resolved through discussion and consensus, with input from a third reviewer when necessary. The selection process was documented using a PRISMA flow diagram. Table 1 displays the search strings specific to the database.

**Table 1:** Database-specific search strings CLP: cleft lip and palate, UCLP: unilateral cleft lip and palate

Database	Search terms
PubMed	))"cleft lip" OR "cleft palate" OR "cleft lip and palate" OR CLP OR UCLP] AND ["Orthodontic Appliances" OR "Orthodontic Retainers" OR "Orthodontics, Corrective" OR "orthodontic treatment" OR Orthodontics OR "orthodontic relapse" OR "compensatory orthodontics"] AND ["Dental Arch" OR "Dental Arches" OR "arch dimension*" OR "arch width" OR "arch length" OR "maxillary dimension*" OR "maxillary arch" OR Maxilla OR "follow up*" OR "follow-up*" OR Intercanine OR "Inter canine" OR "Inter-canine" OR Interpremolar OR "Inter premolar" OR "Inter-premolar" OR Intermolar OR "Inter molar" OR "Inter-molar" OR Relapse OR postretention OR "Post retention" OR "Post-retention" OR posttreatment OR "Post treatment" OR "Post-treatment" OR Retention OR Stability OR "Long-term"((
Web of Science	))“cleft lip” OR “cleft palate” OR “cleft lip and palate”] AND [“Orthodontic Appliances” OR Orthodontics OR “Orthodontics, Corrective” OR “Orthodontic Retainers” OR “orthodontic treatment” OR orthodontic OR “Compensatory treatment” OR “Compensatory orthodontics” OR “Orthodontic camouflage”] AND [“Dental Arch” OR Maxilla OR “Dental Arches” OR “dental arch dimension*” OR “maxillary dimension*” OR Stability OR “Long-term” OR “Long term” OR “Maxillary dental arch*” OR “Maxillary arch form*” OR “Maxillary arch” OR “Alveolar arch” OR “arch width” OR “arch length” OR Maxillary OR “follow up*” OR “follow-up*” OR Intercanine OR “Inter canine” OR “Inter-canine” OR Interpremolar OR “Inter premolar” OR “Inter-premolar” OR Intermolar OR “Inter molar” OR “Inter-molar” OR Relapse OR postretention OR “Post retention” OR “Post-retention” OR posttreatment OR “Post treatment” OR “Post-treatment”((
Scopus	TITLE-ABS-KEY(("cleft lip" OR "cleft palate" OR "cleft lip and palate" OR CLP OR UCLP) AND ("Orthodontic Appliances" OR Orthodontics OR "Orthodontics, Corrective" OR "Orthodontic Retainers" OR "orthodontic treatment" OR "compensatory orthodontics" OR "orthodontic camouflage" OR "orthodontic relapse" OR "orthodontic retention") AND ("Dental Arch" OR Maxilla OR "Dental Arches" OR "dental arch dimension*" OR "maxillary dimension*" OR Stability OR "long-term" OR "long term" OR "maxillary dental arch" OR "maxillary arch form" OR "maxillary arch" OR "alveolar arch" OR "arch width" OR "arch length" OR Maxillary OR "follow up" OR "follow-up" OR Intercanine OR "inter canine" OR "inter-canine" OR Interpremolar OR "inter premolar" OR "inter-premolar" OR Intermolar OR "inter molar" OR "inter-molar" OR Relapse OR "post retention" OR "post-retention" OR posttreatment OR "post-treatment" OR Retention OR "treatment stability"))
Cochrane CENTRAL	(cleft lip OR cleft palate OR cleft lip and palate OR CLP OR UCLP) AND (Orthodontics OR orthodontic treatment OR orthodontic appliances OR orthodontic retainers OR orthodontic retention OR orthodontic relapse OR compensatory orthodontics OR maxillary expansion) AND (Dental Arch OR arch dimension* OR maxillary arch OR Intercanine OR Intermolar OR Stability OR long-term OR follow-up OR Relapse OR Retention)

Data Extraction

Following the selection of eligible studies, two independent reviewers extracted data from the selected studies using standardized extraction forms. Data extraction was performed in duplicate, and disagreements were resolved through consensus discussion or consultation with a third reviewer. Data quality assurance procedures were implemented throughout the extraction process to ensure data integrity. Disagreements regarding extracted values or interpretation were resolved through re-examination of the original publication and discussion with a senior reviewer. All extracted data were entered into electronic databases to facilitate subsequent synthesis and analysis.

Data Items and Outcomes

Extracted data included study design, country, sample size, participant demographics (age and sex), cleft characteristics, orthodontic intervention type and duration, adjunctive procedures, retention protocols, prosthetic rehabilitation modality (where applicable), follow-up duration, and timepoints of outcome assessment. Primary outcomes were quantitative measures of maxillary arch stability, including IC, IM, IPM, and SA. Secondary outcomes were relapse patterns, qualitative clinical stability observations in the absence of numerical data, and adverse event reporting (such as ankylosis, root resorption, or prosthetic complications) when available.

Risk of Bias Assessment

Two independent authors assessed the risk of bias based on the study design. Cohort studies were assessed using the Risk of Bias in Non-randomized Studies of Interventions (ROBINS-I) tool, which evaluates bias across the following seven domains: confounding, selection, classification of interventions, deviations from intended interventions, missing data, measurement of outcomes, and selection of reported results [20]. Case reports were assessed using the JBI checklist for case reports, which evaluates reporting clarity, participant description, diagnostic methods, intervention details, outcome reporting, and adverse event reporting [21].

With respect to the timing of CLP repair, the included studies generally referred to institutional surgical protocols but did not consistently report whether individual patients underwent primary or delayed (secondary) repair relative to the routine timeline. None of the studies stratified stability outcomes according to timing of repair or adjusted for this factor in their analyses. Therefore, any potential influence of late or secondary repair on relapse patterns could not be quantified in this review and should be considered an unmeasured confounder rather than a formally evaluated source of bias.

Assessment of Reporting Bias

Assessment of publication bias, such as funnel plot analysis, was not performed due to the small number of included studies and significant heterogeneity in study designs and outcomes.

Certainty of Evidence

A formal certainty-of-evidence grading assessment was not done due to the limited number of studies and the inclusion of case reports. In addition, there was substantial clinical and methodological heterogeneity, which precluded pooled quantitative synthesis. The three included case reports, by design, provide low-level evidence and were therefore used only to illustrate feasible clinical approaches and to generate hypotheses regarding potential stability patterns, rather than to support any conclusions about the effectiveness of orthodontic treatment stability.

Synthesis of Results

Given the substantial clinical and methodological heterogeneity across the six included studies, quantitative meta-analysis was not feasible. This heterogeneity arose from multiple sources: (1) study design diversity (two prospective cohort studies, one retrospective cohort study, and three case reports); (2) heterogeneous interventions (including SME protocols, RME using mechanical expanders, and comprehensive fixed multibracket appliance therapy); (3) variability in outcome measurement and reporting (absolute arch dimensions at defined time points versus relapse relative to treatment completion, and qualitative reporting in case reports); and (4) variation in follow-up duration and measurement timepoints across studies, which precluded direct quantitative comparison of stability trajectories.

Instead, this review adopted a structured narrative synthesis approach. Descriptive summaries were generated for each study, including study design characteristics, participant demographics, intervention details, and reported outcomes. For quantitative outcomes, tabular summaries of extracted measures (including mean values and standard deviations (SD) where reported) were compiled from studies providing numerical data. When available, additional statistical parameters, such as confidence intervals (CI), were also extracted. Qualitative outcomes from case reports and descriptive sections of cohort studies were synthesized thematically. This structured narrative synthesis ensured transparency while acknowledging the limitations of combining heterogeneous study designs and interventions.

Results

Study Selection

**Figure 1:**
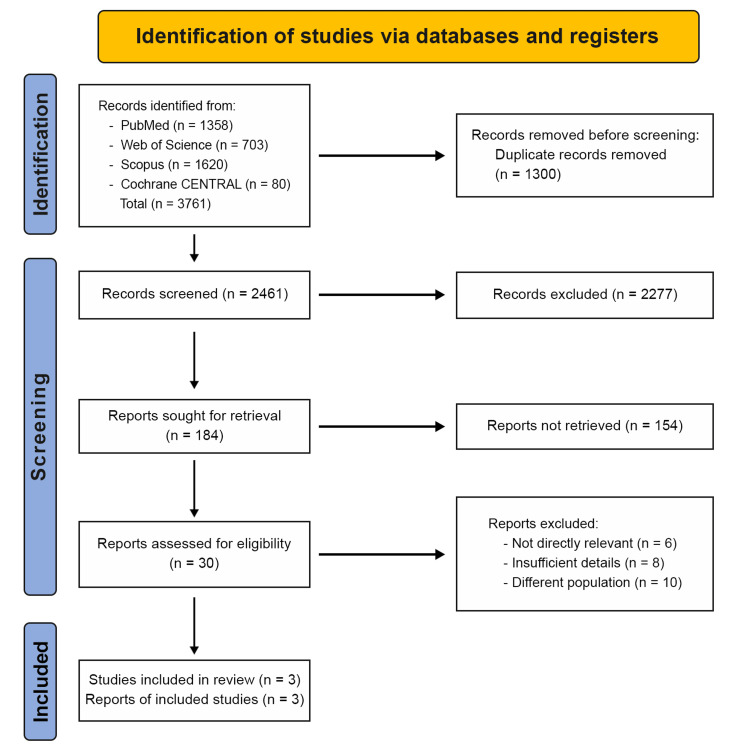
PRISMA flow diagram summarizing the selection process PRISMA: Preferred Reporting Items for Systematic Reviews and Meta-Analyses

The study selection process is summarized in Figure 1. A systematic search of four electronic databases identified 3,761 records (PubMed = 1,358; Web of Science = 703; Scopus = 1,620; Cochrane CENTRAL = 80). After automated deduplication removed 1,300 duplicate records, 2,461 unique records remained for screening. Title and abstract screening by two independent reviewers excluded 2,431 records that did not meet the eligibility criteria, resulting in the selection of 30 reports for full-text review. During full-text review, 24 reports were excluded: five reports did not address orthodontic treatment stability in patients with CLP, eight reports lacked sufficient outcome data such as missing arch-dimension measurements or inadequate follow-up documentation, 10 reports included populations outside the scope of the review including non-cleft populations, syndromic clefts, or cleft patients without orthodontic treatment, and one report evaluated only presurgical orthopedic treatment using an RBJ stimulator in newborns. Six studies consisting of two prospective cohort studies, one retrospective cohort study, and three case reports met the inclusion criteria and were included in the qualitative synthesis, resulting in a total of 130 participants comprising 126 from cohort studies and four from case reports.

Characteristics of Included Studies

The search identified three cohort studies [10,11,20] and three case reports [9,13,21] with a total of 130 participants, comprising 110 participants with UCLP (54 males and 56 females) and 20 non-cleft controls. Participant age ranged from four years eight months to 25 years, and follow-up assessments ranged from nine months (0.75 years) to 9.6 years post-treatment.

The three cohort studies consisted of two prospective studies and one retrospective study. Al-Gunaid et al. [10] conducted a prospective cohort study on 32 Japanese patients (12 males and 20 females) with a mean age of 16.1 ± 3.4 years. All participants had complete UCLP and were stratified by pretreatment maxillary arch form into Group A (symmetrical arch, n = 9), Group B (collapse of minor segment, n = 12), and Group C (collapse of both segments, n = 11). Orthodontic treatment comprised SME combined with edgewise appliances. Follow-up was at least 2 years post-treatment (mean 2.9 ± 2.0 years). Measurements included IC, IPM, and IM using digitized dental casts.

The other prospective study was conducted by Marcusson and Paulin [11] on 39 Swedish patients (25 males and 14 females) with a mean age of 19.1 ± 0.95 years at baseline. All participants had complete UCLP and were divided into three retention groups: no retention (n = 15), bonded twisted retainer extended from canine to canine (n = 13), and onlay or fixed bridge (n = 11). Fixed orthodontic appliances were used for treatment, and the follow-up period was 5.6 years (range 0.9 to 9.6 years). Measurements included IC, inter-second premolar distance (I2PM), IM, and SA using digitized dental casts.

The third included cohort study was done by Soares et al. [22]. This was a retrospective cohort study on 55 participants, comprising 35 individuals with complete UCLP (implant-supported prosthesis group, n = 20; fixed partial denture group, n = 15) and 20 non-cleft controls (n = 20). The implant-supported group had 13 females and seven males (mean age: 25.25 ± 3.2 years), the fixed partial denture group had eight females and seven males (mean age: 25.06 ± 3.36 years), and the control group had 11 females and nine males (mean age: 22.4 ± 4.0 years). All groups received RME followed by comprehensive orthodontic treatment over approximately four years. Follow-up measurements were obtained immediately after orthodontic treatment (T1) and one year after prosthetic rehabilitation (T2) for the cleft groups, and one year after orthodontic completion for the controls. Three-dimensional stereophotogrammetry was used to obtain measurements of intercanine distance, intermolar distance, and arch length. The characteristics of the included cohort studies are summarized in Table 2.

**Table 2:** Characteristics of the included cohort studies SD: standard deviation; NA: not available; UCLP: unilateral cleft lip and palate; IC: intercanine distance; IM: intermolar distance; IPM: interpremolar distance; I2PM: inter-second premolar distance; SA: sagittal arch length; FPD: fixed partial denture; 3D: three-dimensional

Study	Study design	Age, years, mean ± SD	Sample (gender, N)	Setting	Type of CLP	Lip closure	Palate closure	Orthodontic appliance	Collapse form	Retaining	Duration of treatment
Al-Gunaid et al., 2008 [10]	Prospective cohort	16.1 ± 3.4	32 (12 male, 20 female)	Division of Orthodontics, Niigata University Medical and Dental Hospital, Japan	Complete UCLP (10 right; 22 left)	Done between 3 and 12 months (Cronin technique and Millard technique)	Done between 10 and 24 months (push-back method)	Slow maxillary expansion; edgewise appliance	Group A: symmetrical (n = 9); Group B: minor segment collapse (n = 12); Group C: both segments collapse (n = 11)	Bonded retainers (canine-canine) for 2 patients/group; remaining 26 circumferential retainers	At least 2 years
Marcusson and Paulin, 2004 [11]	Prospective cohort	19.1 ± 0.95	39 (25 male, 14 female)	University Hospital, Linköping, Sweden	Complete UCLP (n = 39; laterality not separately reported)	Done at 3 months (Millard technique)	Done at 18 months (Wardill-Kilner technique)	Fixed orthodontic appliance	NA	Bonded twist retainer (canine-canine) (n = 13); onlay/fixed bridge (n = 11); no retention (n = 15)	The follow-up was 5.6 years between baseline and follow-up
Soares et al., 2022 [22]	Retrospective cohort	25.25 ± 3.2 (implant); 25.06 ± 3.36 (FPD); 22.4 ± 4 (control)	55 (23 male, 32 female)	Bauru School of Dentistry, University of São Paulo (USP), and Hospital for Rehabilitation of Craniofacial Anomalies, University of São Paulo (USP), Bauru, Brazil	Complete UCLP (cleft groups)	NA	NA	Rapid maxillary expansion; orthodontic treatment	NA	Implant-supported prosthesis (n = 20); fixed partial denture (n = 15); non-cleft controls (n = 20)	T1: immediately after orthodontic treatment; T2: 1 year after prosthetic rehabilitation (cleft) or 1 year after orthodontic completion (controls)

Three case reports were also included to describe additional orthodontic treatment approaches and stability outcomes in complex unilateral CLP presentations. The case reports used diverse treatment approaches and outcomes. Dhole et al. [9] reported a 10-year-10-month-old female with unilateral complete CLP treated with two-phase orthodontic therapy, with stable outcomes at one-year follow-up. Souza et al. [13] documented a nine-year-old male with unilateral CLP and canine-first premolar transposition treated with slow maxillary expansion, secondary alveolar bone grafting, and comprehensive fixed appliance therapy incorporating mini-implants, with stability at three-year follow-up. Tanimoto et al. [23] reported two male cases of unilateral CLP (aged four years eight months and nine years three months) treated with fixed appliances, followed by tooth transplantation with autogenous bone grafting, with acceptable outcomes and stable transplanted teeth without evidence of ankylosis or root resorption. A summary of the included case reports is presented in Table 3.

**Table 3:** Summary of Included Case Reports CLP: cleft lip and palate; MBT: McLaughlin, Bennett, and Trevisi

Study	Study design	Age	Gender	CLP	Orthodontic treatment	Orthodontic treatment outcomes and results	Orthodontic treatment conclusions
Dhole et al., 2017 [9]	Case report	10 years 10 months	Female	Unilateral complete CLP on the left side	Palatal expander (Leone, Florence). Reverse pull headgear (Petit design, Ormco). Fixed appliances (0.022 MBT, 3M Unitek, Gemini)	Class III incisor relation: Improved to Class I. Overjet: Increased from -3 mm to 1.5 mm. Overbite: Decreased from 2 mm to 1.5 mm. Crossbite: Corrected. Skeletal class III: Improved. V-shaped upper arch: Expanded and corrected	The orthodontic treatment was successful in addressing the patient's dental and facial concerns. The patient achieved a more balanced occlusion, improved facial profile, and corrected skeletal class III relationship
Souza et al., 2020 [13]	Case report	9 years	Male	Unilateral CLP	Slow maxillary expansion. Fixed appliances. Mini-implant. Extraction (right maxillary second premolar). Mesialization and tooth-position adjustments (bending and rebonding)	The treatment showed significant improvement in facial aesthetics, showing a symmetrical appearance. The dental occlusion was improved from a Class II molar relationship, anterior and posterior crossbite, absence of left maxillary lateral incisor, to Class I molar and canine relationships, good intercuspation, adequate overjet and overbite, and the space of the missing lateral incisor was closed	The orthodontic treatment was successful in correcting the facial asymmetry caused by the cleft, establishing a balanced dental occlusion, improving the skeletal pattern, and enhancing the overall aesthetics and function of the bite
Tanimoto et al., 2010 [23]	Case report 1	4 years 8 months	Male	Unilateral CLP on the left side	Fixed appliances. Lingual arch, lateral expansion appliances, and multi-bracket appliances	Acceptable occlusion achieved, transplanted upper premolar stable, no bone ankylosis or root resorption	Both cases demonstrated successful orthodontic treatment outcomes, with the correction of crossbites, resolution of dental anomalies, and stable transplanted teeth
	Case report 2	9 years 3 months	Male	Unilateral CLP on the left side	Fixed appliances. Lingual arch, quad-helix appliances, and multi-bracket appliances	Not reported separately	

Risk of Bias Assessment

Risk of bias for cohort studies was assessed using the ROBINS-I tool [20]. Two of the three cohort studies (Al-Gunaid et al., 2008; Soares et al., 2022) demonstrated an overall moderate risk of bias [10,22]. In contrast, Marcusson and Paulin (2004) demonstrated an overall serious risk of bias, primarily driven by confounding and deviations from intended interventions (retention type not adjusted for, lack of compliance documentation, and variability in follow-up not addressed) [11]. Detailed domain-level judgments are presented in Table 4.

**Table 4:** Risk of bias assessment of the included cohort studies using the ROBINS-I tool ROBINS-I: Risk of Bias in Non-randomized Studies of Interventions; Overall: overall risk-of-bias judgment

Study	D1: confounding bias	D2: selection bias	D3: bias in the classification of interventions	D4: bias due to deviation from intended interventions	D5: bias due to missing data	D6: bias in the measurement of the outcome	D7: bias in the selection of the reported result	Overall
Al-Gunaid et al., 2008 [10]	Moderate	Low	Low	Moderate	Low	Moderate	Low	Moderate
Marcusson and Paulin, 2004 [11]	Serious	Moderate	Low	Serious	Moderate	Low	Low	Serious
Soares et al., 2022 [22]	Moderate	Low	Low	Moderate	Low	Low	Low	Moderate

**Figure 2:**
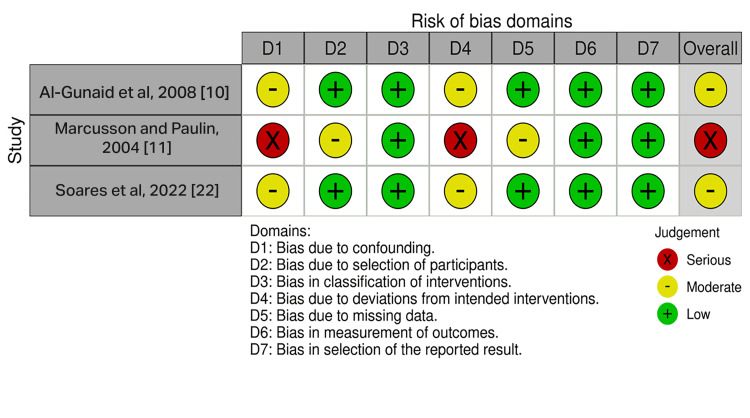
Risk of bias traffic light plot for cohort studies (ROBINS-I) Green: low risk of bias; yellow: moderate risk of bias; red: serious risk of bias ROBINS-I: Risk of Bias in Non-randomized Studies of Interventions

A ROBINS-I traffic light plot summarizing domain-level judgments across the included cohort studies is shown in Figure 2. The plot was generated using the robvis visualization tool [24].

For the three case reports, the risk of bias was assessed using the JBI checklist for case reports [21]. While the included case reports generally demonstrated adequate reporting across most JBI domains, adverse event reporting was incomplete in two reports, namely Dhole et al. (2017) and Tanimoto et al. (2010) [9, 23]. This limits the interpretability of harms and is a common limitation of case report evidence. The detailed JBI checklist assessment is shown in Table 5.

**Table 5:** Risk of bias assessment using the JBI checklist for case reports Responses were recorded as Yes, No, Unclear, or Partial (partially reported)

Study	1. Were the patient’s demographic characteristics clearly described?	2. Was the patient’s history clearly described and presented as a timeline?	3. Was the current clinical condition of the patient on presentation clearly described?	4. Were diagnostic tests or assessment methods and the results clearly described?	5. Was the intervention or treatment procedure clearly described?	6. Was the post-intervention clinical condition clearly described?	7. Were adverse events, harms, or unanticipated events identified and described?	8. Does the case report provide takeaway lessons?
Dhole et al., 2017 [9]	Yes	Yes	Yes	Yes	Yes	Yes	Partial	Yes
Souza et al., 2020 [13]	Yes	Yes	Yes	Yes	Yes	Yes	Yes	Yes
Tanimoto et al., 2010 [23]	Yes	Yes	Yes	Yes	Yes	Yes	Partial	Yes

Orthodontic Treatment Stability

As shown in Table 6, three studies evaluated post-treatment stability of the maxillary arch. Substantial heterogeneity in relapse patterns was observed across study populations and retention protocols. The primary outcomes assessed were IC, IPM/I2PM, IM, and SA. Overall, orthodontic treatment was associated with measurable changes in dental arch dimensions, with cleft groups generally demonstrating narrower arches than non-cleft controls. However, post-treatment relapse was reported across multiple measurement parameters, signifying the potential influence of retention protocols and the challenge of maintaining orthodontic correction in this population.

**Table 6:** Mean values for orthodontic treatment stability (maxillary arch) SD: standard deviation; NA: not available; IC: intercanine distance; IM: intermolar distance; IPM: interpremolar width; I1PM: inter first premolar width; I2PM: inter second premolar width; SA: sagittal arch length; FPD: fixed partial denture; 3D: 3-dimensional

Study	Parameter	IC, mean ± SD	IPM, mean ± SD	IM, mean ± SD	SA, mean ± SD	Conclusion
Al-Gunaid et al., 2008 [10]	Collapse of minor segment	−0.54 ± 0.55	−0.26 ± 0.7	−0.03 ± 0.85	NA	The symmetrical arch showed stable results in all measurements. Patients with collapse of a minor segment showed posttreatment relapse in the IC width only, whereas patients with collapse of both minor segments demonstrated significant posttreatment relapses in the IPM and IM widths
	Collapse of both segments	−0.12 ± 0.99	−1.19 ± 1	−1.14 ± 0.48	NA	
	Symmetrical arch form	−0.62 ± 0.90	−0.69 ± 1.04	−0.22 ± 0.63	NA	
Marcusson and Paulin, 2004 [11]	Retention with onlay or fixed bridge	−0.5 ± 0.8	−1.7 ± 1.4	−1.4 ± 1.3	−0.6 ± 0.9	There was a significant reduction in the dimensions of the maxillary arch in relation to the total sample, regardless of the use of an orthodontic retainer or prosthesis
	Retention with a bonded retainer	−0.6 ± 0.6	−1.7 ± 1.6	−1.1 ± 1.3	−0.6 ± 0.8	
	No retention	−0.7 ± 0.9	−1.6 ± 1.4	−1.7 ± 1.6	−1 ± 0.9	
Soares et al., 2022 [22]	Dental implant	−0.61 ± 1.91		−0.91 ± 1.68	−0.54 ± 1.65	FPD may provide better stability of the orthodontic outcomes than an implant-supported prosthesis. Greater instability occurred at the molar area
	FPD	0.58 ± 1.95		−1.84 ± 2.01	0 ± 1.12	
	Non-cleft	0.01 ± 0.76		−0.37 ± 0.66	−0.32 ± 0.89	

Intercanine Distance

Intercanine distance was measured in all three cohort studies. Marcusson and Paulin documented that patients without maxillary retention exhibited the greatest mean relapse (-0.7 ± 0.9 mm), whereas those retained with bonded retainers (-0.6 ± 0.6 mm) or onlays or fixed bridges (−0.5 ± 0.8 mm) demonstrated comparatively smaller mean reductions [11]. Al-Gunaid et al. reported that patients with a symmetrical arch form had minimal mean relapse (−0.62 ± 0.90 mm), whereas those with collapse of the minor segment exhibited moderate mean relapse (−0.54 ± 0.55 mm). Patients with collapse of both maxillary segments showed a smaller mean change (−0.12 ± 0.99 mm), although the large standard deviation indicates substantial variability [10]. Soares et al. reported a mean reduction in the implant-supported group (−0.61 ± 1.91 mm), a mean increase in the fixed partial denture group (0.58 ± 1.95 mm), and minimal change in the non-cleft control group (0.01 ± 0.76 mm) [22].

Interpremolar Distance

Interpremolar distance measurements suggested greater relapse in patients with more severe arch collapse. Al-Gunaid et al. reported the greatest mean relapse (−1.19 ± 1.00 mm) in patients with collapse of both segments, compared with smaller mean changes in patients with collapse of the minor segment only (−0.26 ± 0.70 mm) and those with a symmetrical arch form (−0.69 ± 1.04 mm) [10]. Marcusson and Paulin documented comparable mean inter-second premolar relapses across retention groups, including no retention (−1.6 ± 1.4 mm), bonded retainers (−1.7 ± 1.6 mm), and onlay or fixed bridge retention (−1.7 ± 1.4 mm) [11]. Collectively, these findings indicate that posterior arch stability may remain a challenge despite different retention modalities. Soares et al. did not report premolar-width outcomes in their three-dimensional measurement protocol [22].

Intermolar Distance

Intermolar distance was measured in three studies and generally showed substantial relapse. Marcusson and Paulin reported the largest mean reduction in the no-retention group (−1.7 ± 1.6 mm), followed by the onlay or fixed bridge group (−1.4 ± 1.3 mm) and the bonded retainer group (−1.1 ± 1.3 mm) [11]. Conversely, Al-Gunaid et al. found that relapse varied by arch form classification, with minimal mean relapse in symmetrical arches (−0.22 ± 0.63 mm) and greater mean relapse in arches with collapse of both segments (−1.14 ± 0.48 mm) [10]. Soares et al. reported a greater mean reduction in the fixed partial denture group (−1.84 ± 2.01 mm) than in the implant-supported group (−0.91 ± 1.68 mm), with a smaller mean change (−0.37 ± 0.66 mm) in the non-cleft controls [22]. Overall, these findings suggest that the posterior region of the maxillary arch may be particularly susceptible to post-treatment instability.

Sagittal Arch Length

Sagittal arch length was measured in two of the included studies. Marcusson and Paulin reported the greatest mean relapse (−1.0 ± 0.9 mm) in patients without retainers, whereas patients with bonded retainers or onlays, or fixed bridges showed smaller mean reductions (−0.6 ± 0.8 mm to −0.6 ± 0.9 mm) [11]. Soares et al. reported a mean reduction of −0.54 ± 1.65 mm in the implant-supported group [22].

Individual Case Reports

Dhole et al. reported orthodontic treatment of a 10-year-10-month-old female with unilateral complete cleft lip and palate. Treatment improved the molar relationship from Class III to Class I, with overjet improving from -3 mm to 1.5 mm and overbite reducing from 2 mm to 1.5 mm. In addition, treatment also corrected the crossbite, improved the V-shaped upper arch, and improved the skeletal Class III relationship [9].

Souza et al. described the orthodontic management of a nine-year-old male with unilateral cleft lip and palate and maxillary canine-first premolar transposition. A combination of fixed appliances, mini-implant anchorage, and tooth extraction led to improvement of the molar relationship from Class II to Class I and resolution of anterior and posterior crossbites. Space closure for the missing lateral incisor was also achieved, and the outcomes were stable at the three-year follow-up appointment [13].

Tanimoto et al. presented two cases of unilateral cleft lip and palate in male children aged four years eight months and nine years three months treated with fixed appliances incorporating lingual arches and expansion appliances, followed by tooth transplantation with autogenous bone grafting [23]. In the first patient (three-year treatment), anterior and posterior crossbites were corrected, and the transplanted premolar remained stable without ankylosis or root resorption. In the second patient (two-year treatment), the anterior crossbite was corrected, and the transplanted premolar remained stable without major complications. Across both cases, the transplanted teeth were reported to be stable with no evidence of bone ankylosis or root resorption [23].

Discussion

This systematic review of six observational studies (three cohort studies and three case reports) included 130 participants, comprising 110 participants with UCLP (54 males and 56 females) and 20 non-cleft controls. Fixed appliance therapy, the most commonly reported orthodontic approach, led to improvements in dental occlusion and facial aesthetics across the included evidence. Despite variations in treatment protocols, all interventions addressed core cleft-related challenges, including maxillary arch constriction, dental malalignment, and the tendency toward Class III skeletal relationships. However, maintaining long-term post-treatment stability remained a consistent clinical challenge across studies.

Post-Treatment Stability and Relapse

Across the included cohort evidence, a general tendency toward post-treatment constriction of maxillary arch dimensions was observed, particularly in transverse measures, and sagittal arch length reduction was also reported where assessed. Al-Gunaid et al. suggested that pretreatment maxillary arch form may influence post-treatment stability, as patients with collapsed arch segments showed greater relapse tendencies than those with symmetrical arch forms. In their cohort, collapse of the minor segment was associated mainly with relapse in intercanine distance, whereas collapse of both segments was associated with greater relapse in interpremolar and intermolar dimensions [10]. These findings suggest that the arch morphology before treatment may act as a practical prognostic indicator when planning retention strategies. None of the included cohort studies, however, systematically evaluated or stratified relapse according to systemic conditions (such as underlying medical comorbidities, growth or nutritional status, or other general health factors). Relapse was reported almost exclusively in relation to local dental and craniofacial variables, including arch form, expansion protocol, and retention strategy. Therefore, any potential contribution of systemic conditions to post-treatment instability remains unclear within the available evidence and should be considered a possible unmeasured confounder rather than an assessed determinant of relapse.

Variation in Relapse Patterns

The included studies indicated that relapse patterns may differ depending on the region of the arch. In general, posterior transverse stability appeared more challenging to maintain than anterior dimensions in several groups. Soares et al. compared fixed partial denture and implant-supported prosthetic rehabilitation in adults and reported better anterior stability in the fixed partial denture group, consistent with a potential splinting effect across the anterior cleft site. However, greater instability was still observed in the intermolar region, indicating that anterior stabilization alone may not be sufficient to prevent posterior transverse relapse [22]. In Al-Gunaid et al., the symmetrical arch form group demonstrated relatively small mean changes in intermolar width (−0.22 ± 0.63 mm), while greater intermolar relapse was observed in the collapse of both segments group (−1.14 ± 0.48 mm) [10]. Collectively, these findings indicate the posterior maxillary arch as a key area of vulnerability for relapse and suggest that retention planning should address posterior transverse stability rather than focusing only on the anterior segment.

Maxillary expansion protocols, whether slow or rapid, may also influence long-term transverse stability, although direct comparative conclusions are limited by heterogeneity in appliances, timing, and outcome reporting. Given the potential for posterior relapse, long-term retention and structured follow-up are clinically relevant to detect early relapse and allow timely occlusal adjustments where needed. Some external evidence has also suggested that prolonged retainer wear may be associated with improved stability outcomes, although this should be interpreted as a supportive background finding rather than direct evidence from the included studies [25]. From a clinical standpoint, relapse in these studies was typically identified through progressive narrowing of the maxillary arch, reduction in intermolar and intercanine widths, recurrence of anterior or posterior crossbites, reopening of spaces in the cleft region, rotation or migration of teeth adjacent to the cleft, and loss of posterior occlusal intercuspation. These features reflect a gradual loss of transverse expansion and indicate the need for closer monitoring and reinforcement of retention strategies.

Effectiveness of Case-Based Interventions

The three case reports illustrate the applicability of orthodontic management in complex unilateral cleft presentations, producing improvements in occlusion and facial aesthetics with individualized treatment planning. Souza et al. documented treatment with a mini-implant anchorage and reported root apical resorption monitoring with preservation of tissue health. The case report also provided useful safety-related details [13]. Dhole et al. described combined orthopedic and orthodontic approaches alongside surgical interventions, illustrating the complexity of treatment sequences in cleft care [9]. Tanimoto et al. reported stable transplanted teeth with no evidence of ankylosis or root resorption after treatment in the cleft region, although broader adverse event surveillance was limited [23]. Overall, these case reports are best interpreted as descriptive evidence supporting the feasibility of orthodontic treatment and the importance of clinical decision-making rather than definitive efficacy or safety evidence.

Limitations and Evidence Quality

This review has important limitations. The methodological quality of the included cohort studies was found to have a moderate to serious risk of bias, driven largely by potential confounding and incomplete documentation of intervention adherence. Furthermore, there was substantial heterogeneity across studies due to varying treatment modalities, retention protocols, patient age, follow-up duration, and measurement methods; this precluded quantitative meta-analysis and limited the strength of comparative clinical conclusions. The inclusion of case reports further reduces certainty, as these designs have inherent limitations in generalizability and are susceptible to selection and reporting bias. Additionally, most evidence originated from settings with established multidisciplinary cleft services, which may limit the transferability of findings to other locations. Finally, key confounders such as cleft anatomy severity, timing and technique of alveolar bone grafting, and timing of prosthetic rehabilitation were not consistently reported or adjusted for across studies. Taken together, these factors may either exaggerate the apparent stability of treatment (if high-risk or poorly retained cases were underreported) or underestimate it (if more complex, relapse-prone cases were disproportionately represented). As a result, the true magnitude of relapse in clinical practice remains uncertain and must be interpreted with caution.

Clinical Implications

Despite these limitations, several clinically relevant points emerge. The maxillary arch form before treatment appears to be a useful indicator for relapse risk and may help guide retention intensity and duration. Furthermore, retention strategies should consider the entire maxillary arch, with specific attention to posterior transverse stability. Prosthetic splinting may support anterior stability but does not necessarily prevent posterior relapse, suggesting that orthodontic retention and prosthetic rehabilitation should be viewed as complementary rather than interchangeable approaches.

These findings primarily apply to non-syndromic children, adolescents, and young adults with complete unilateral cleft lip and palate who are treated in multidisciplinary, specialized cleft centers using contemporary orthodontic protocols, including expansion appliances and fixed multibracket systems. Because most included studies were conducted in highly specialized settings with structured follow-up and individualized retention programs, the ability to generalize these results to other patient populations, treatment protocols, or less specialized environments is limited. Finally, long-term monitoring remains essential to identify relapse early and help maintain functional occlusion.

Recommendations for Future Research

Future research should now move toward clearer, clinically focused priorities. First, there is a need for prospective longitudinal studies that use standardized and reproducible outcome measures for maxillary stability, particularly intercanine, interpremolar, and intermolar widths, so that results can be compared across studies. Second, because the posterior maxillary arch appears to be the most vulnerable region for relapse, future research should specifically investigate strategies aimed at improving posterior transverse stability, including the role of expansion protocols and prosthetic splinting. Third, long-term retention should be systematically documented, with careful monitoring of adherence, duration of wear, and the relationship between compliance and relapse. Addressing these priorities will allow future evidence to more clearly identify which stability strategies are truly effective in patients with unilateral cleft lip and palate.

## Conclusions

This systematic review demonstrates that orthodontic treatment can improve dental occlusion and facial aesthetics in individuals with CLP; however, long-term post-treatment stability remains a persistent clinical challenge. Within the limits of three observational cohort studies, including one with a serious risk of bias, and three descriptive case reports, relapse patterns appear to vary according to pretreatment arch morphology and the specific region of the maxillary arch, with posterior transverse dimensions showing particular susceptibility in several studies. These findings suggest, rather than conclusively establish, the importance of using retention strategies that address the entire maxillary arch and the need for long-term follow-up to detect relapse and enable prompt occlusal adjustments if required. Nevertheless, the limited number of included studies, the inclusion of case reports, and considerable clinical and methodological heterogeneity limit the strength of definitive clinical recommendations. High-quality prospective research with standardized outcome measures and extended follow-up is needed to establish clearer guidance on optimal long-term stability protocols for the UCLP patient population.
